# The Endocannabinoid System: A Potential Therapeutic Target for Coagulopathies

**DOI:** 10.3390/metabo12060541

**Published:** 2022-06-14

**Authors:** Wujood Khayat, Christian Lehmann

**Affiliations:** 1 Department of Physiology and Biophysics, Dalhousie University, Halifax, NS B3H 4R2, Canada; 2 Department of Basic Medical Sciences, College of Medicine, King Saud bin Abdulaziz University for Health Sciences, Jeddah 21423, Saudi Arabia; 3 Department of Pharmacology, Dalhousie University, Halifax, NS B3H 4R2, Canada; 4 Department of Anesthesia, Pain Management and Perioperative Medicine, Dalhousie University, Halifax, NS B3H 4R2, Canada

**Keywords:** cannabinoids, endocannabinoids, endocannabinoid system, marijuana, cannabis, coagulopathy, blood coagulation, thrombosis, hemorrhage

## Abstract

Abnormal blood coagulation or coagulopathy is a common manifestation of many pathological conditions. It occurs when there is an imbalance between the activities of the coagulation system and the fibrinolytic system, leading to excessive or impaired intravascular blood clot formation, which can disturb blood flow causing ischemia or hemorrhage in the affected tissues. A growing body of evidence has demonstrated blood coagulation abnormalities in association with cannabinoid use, suggesting the involvement of the endogenous cannabinoid system (ECS) in modulating blood coagulation. However, the evidence in the literature has been controversial on whether cannabinoids promote or inhibit blood coagulation. The ECS has been extensively studied in recent years for its potential as a therapeutic target for many diseases. This review provides a brief introduction to the ECS and discusses the reported anticoagulatory and procoagulatory effects of various cannabinoids, highlighting some possible mechanisms that might underlie the observed effects. Understanding the coagulatory effects of cannabinoids and the interaction between the coagulation system and the ECS is vital for developing novel therapeutics for coagulopathies.

## 1. Introduction

The endogenous cannabinoid system (ECS) is a complex regulatory network involved in the homeostasis of the organism at the cell, tissue, and organ levels. It is involved in embryogenesis and neurodevelopment [1,2], neuromodulation and neuroprotection [3,4], learning and memory [5,6], motor control [7], pain modulation [8,9], metabolic and immune responses [10,11,12], autonomic regulation of organ functions [13,14,15], among others. Given its involvement in tuning a wide range of pathophysiological processes, the ECS has attracted many researchers to study its potential as a therapeutic target in many pathological conditions such as cancer, cardiovascular disease, neurological disorders, inflammatory conditions, obesity, and metabolic disorders [11,16,17,18].

The versatile ECS consists primarily of (i) cannabinoid receptors type 1 and 2 (CB1 and CB2); (ii) their endogenous activating ligands including arachidonoyl ethanolamide (AEA), also known as anandamide, 2-arachidonoylglycerol (2-AG), virodhamine (O-arachidonoyl ethanolamine (O-AEA), and N-Arachidonoyl Dopamine [19], the latter two being sometimes considered as endocannabinoid-like ligands; and (iii) the enzymes involved in the metabolism of these endocannabinoids, such as diacylglycerol lipase (DAGL), N-arachidonoyl phosphatidylethanolamine phospholipase D (NAPE-PLD), monoacylglycerol lipase (MAGL), and fatty acid amide hydrolase (FAAH) [20,21]. Both CB1 and CB2 receptors belong to the class A subfamily of G protein-coupled receptors (GPCRs). Activation of these receptors results in cellular signaling that (i) inhibits adenylyl cyclase causing a decrease in intracellular cyclic adenosine monophosphate (cAMP) and protein kinase A levels, (ii) inhibits voltage-dependent N and P/Q type Ca^2+^ channels leading to a decrease in calcium influx, (iii) stimulates type A K^+^ channels causing an increase in potassium efflux, and (iv) stimulates mitogen-activated protein kinases (MAPK) [22]. The best characterized endogenous ligands of CB1/CB2 receptors, AEA and 2-AG, are eicosanoids that are synthesized on demand from phospholipid precursors. NAPE-PLD catalyzes the synthesis of AEA [23], while DAGL is involved in 2-AG synthesis [24]. AEA and 2-AG have a short activity in vivo that is followed by rapid cellular uptake and intracellular degradation into arachidonic acid (AA) through metabolic reactions facilitated by the catalytic activities of FAAH and MAGL, respectively [25,26]. With the activities of various enzymes, AA is further metabolized into other eicosanoids, such as thromboxane A_2_ (TXA_2_) and prostaglandins [27]. AEA can act as a partial or full agonist on CB1 receptors depending on the system, but it has low efficacy at CB2 receptors, whereas 2-AG is considered a full agonist for both receptors [28]. In addition to the endogenous ligands of CB1 and CB2, these receptors can be modulated exogenously by other compounds including various synthetic receptor agonists and antagonists, as well as by naturally occurring plant-derived cannabinoids (phytocannabinoids), such as Δ^9^-tetrahydrocannabinol (Δ^9^-THC), cannabinol (CBN), and cannabigerol (CBG). Δ^9^-THC represents the most abundant cannabinoid in marijuana and is considered the main constituent responsible for its psychoactive effects [29,30].

CB1 receptors are widely distributed throughout the body, but an abundant tissue expression has been reported in regions of the central nervous system including basal ganglia, hippocampus, cerebellum, amygdala, cingulate cortex, medial hypothalamus, and spinal cord [31]. Peripheral CB1 tissue expression is found at lower, yet functional levels in postganglionic autonomic nerve terminals [28], vascular endothelial and smooth muscle cells [32], myocardium [33], skeletal muscle [34,35], liver [36], and adipose tissue [16]. By contrast, CB2 receptors are more limited to peripheral tissues with a predominant expression in immune and hematopoietic cells [22,37], although recent findings suggest the expression of CB2 receptors centrally in neurons and glia under pathological conditions [38,39,40,41]. Changes in the expression and/or function of CB1/CB2 receptors indicate an impaired endocannabinoid tone and have been detected in a great number of disorders, making the ECS a potential therapeutic target for many diseases [17,21,42].

Hemostasis is a tightly regulated physiological process that is achieved by a delicate balance between the activities of the coagulation system and the fibrinolytic system to ensure normal blood circulation. Blood coagulation disorders or coagulopathies are a group of disorders in which there is a hypercoagulative state leading to excessive abnormal blood clot formation and tissue ischemia, or hypocoagulation with impaired blood clot formation or extensive blood clot lysis, both of which can result in hemorrhage [43]. Accumulating evidence in the literature points to links connecting cannabinoid actions with blood coagulation abnormalities, suggesting crosstalk between the coagulation system and the ECS. However, the evidence has been conflicting, and it remains unclear whether cannabinoids have procoagulatory or anticoagulatory effects. Understanding the nature of the cannabinoid coagulatory effects as well as the interaction between the coagulation system and the ECS is essential in utilizing the ECS as a potential target for developing novel therapeutics for coagulopathies. This paper aims to review the potential anticoagulatory and procoagulatory effects of various cannabinoids, highlighting the possible mechanisms that might underlie some effects of these cannabinoids on blood coagulation. To facilitate reading the review, the reported anticoagulatory effects of (i) synthetic, (ii) plant-derived, and (iii) endogenous cannabinoids will be presented first, followed by their reported procoagulatory effects. Finally, discussing these findings with a reflection on the potential clinical uses of cannabinoids in modulating blood coagulation will be presented.

## 2. Anticoagulatory Effects of Cannabinoids

Anticoagulatory effects of cannabinoids have been reported in a few preclinical studies and clinical case reports. Recently, in 2018, a multistate outbreak of synthetic cannabinoid-associated coagulopathy was declared in the Midwest of the United States with a series of case reports indicating an association between the (over-)use of synthetic cannabinoids and the incidence of coagulopathic hemorrhage, which may suggest potential anticoagulatory effects of these cannabinoids. However, in many of these cases, the hemorrhage induced by synthetic cannabinoid consumption seems to be an indirect effect and attributed to contamination of these cannabinoids with Brodifacoum, a commonly used rodenticide that functions as a long-acting vitamin K-dependent antagonist, hence, has anticoagulatory properties [44,45,46,47,48]. In other cases, however, the reported coagulopathic hemorrhage was thought to be a result of drug–drug interaction. Patients were on anticoagulative warfarin therapy and presented to clinics with events of supratherapeutic INR with or without bleeding in association with therapeutic or recreational uses of phytocannabinoids. It has been suggested that these cannabinoids may potentiate the anticoagulative effect of warfarin through cytochrome P450 interaction, thus, close monitoring and adjusting of warfarin doses are necessary for patients who consume cannabinoids [49,50,51].

Some preclinical studies, however, have indeed demonstrated anticoagulatory effects of phytocannabinoids as well as endocannabinoids. Levendal and Frost [12] studied the metabolic and coagulatory effects of a plant-derived cannabinoid, organic *Cannabis sativa* L. extract, administered subcutaneously, in streptozocin-induced diabetic Wistar rats. Thrombin-induced clotting time showed a significant prolongation in cannabis-treated diabetic rats compared to a vehicle. A similar effect of the cannabis extract on thrombin-induced clotting time was observed in rats without diabetes compared to the vehicle group.

In another study conducted by the same group [52], researchers have extended their studies to examine the possible pro-/anti-coagulatory effects of cannabis extract, and three other major phytocannabinoids including THC, CBN, and CBD, on thrombin activity, using both in vitro and in vivo approaches. To evaluate the in vitro effects, samples of human plasma were treated with saline, cannabis extracts, or phytocannabinoids and a thrombin assay was used to monitor the clotting induced by adding bovine thrombin. While CBD did not show any significant effect on thrombin activity, the other cannabinoids exhibited various degrees of significant inhibition with a 5-fold greater inhibition for THC compared to that of CBN. Furthermore, the IC_50_ values were determined to be 1.79 mg/mL and 9.89 mg/mL for THC and cannabis extract, respectively, whereas no sufficient inhibition was produced by CBN for an IC_50_ value to be determined. Utilizing a turbidimetric clotting assay, CBD had no significant inhibition on thrombin-induced clotting formation, while both THC and CBN showed significant and comparable inhibition with IC_50_ values of 87 µg/mL and 83 µg/mL, respectively. Cannabis extract also significantly inhibited thrombin-induced clotting, but with a greater IC_50_ value of 0.6 mg/mL. The in vivo coagulatory effect of cannabis extract was evaluated on lean and obese rats. The experimental groups of lean (LE) and obese (OE) rats were treated every alternate day with subcutaneous injections of cannabis extract for 28 days, whereas the control groups (LC and OC) received a vehicle. It has been shown that OC significantly had a 1.7-fold shorter 50% clotting time than that of LC, suggesting that obesity leads to a procoagulatory state. LE had a 1.5-fold longer 50% clotting time compared to LC. The anticoagulatory effect of cannabis extract was even more prominent in obese rats, as OE had a 2-fold longer 50% clotting time compared to OC. Collectively, the results of these studies demonstrate that cannabis extract, THC, and CBN inhibit plasma clot formation both in vitro and in vivo and, thus, display an anticoagulatory activity.

The coagulatory effects of the phytocannabinoids olivetol, CBG, CBD, CBN, and THC were also examined on human and rabbit platelets [53]. It has been found that ADP-induced platelet aggregation was inhibited by all these cannabinoids in a dose-dependent manner both in human and rabbit platelets. A partial primary inhibition and a total secondary inhibition by these compounds were observed in human platelet aggregation induced by adrenaline. These cannabinoids also showed a dose-dependent inhibition of rabbit platelet aggregation when the aggregation was induced by PAF. Although these cannabinoids had inhibitory effects on human and rabbit platelet aggregation, their effects on serotonin [^I4^C]5-HT release did not correlate with the inhibition of aggregation. 

In addition to the anticoagulatory effects observed with the abovementioned plant-derived cannabinoids, diffuse alveolar hemorrhage and hemoptysis have also been linked to cannabis smoking [54,55,56], which may suggest an anticoagulatory activity of some components of cannabis. In these cases; however, the exact contents of the smoked cannabis are unknown, and the underlying mechanisms of the bleeding remain to be elucidated. 

The endocannabinoid AEA has also been shown to have an anticoagulatory effect. In a study conducted by De Angelis et al. [57] to examine the role of AEA on platelet function both in vitro and ex vivo, it has been found that platelet aggregation in suspension and α-granule release induced by collagen, collagen-derived peptide (CRP-XL), ADP, AA, and TXA_2_ analog, U46619, were prevented by AEA, which inhibits the expression of platelet activation surface marker P selectin. Stimulating platelet aggregation with collagen or CRP-XL, but not with the other agonists, significantly impaired calcium mobilization; however, collagen-induced phosphorylation of spleen tyrosine kinase (Syk) was inhibited by AEA. Furthermore, treating platelets with AEA results in limiting glycoprotein IIb/IIIa activation and, thus, reducing platelet spreading on immobilized fibrinogen, decreasing platelet capacity for binding fibrinogen in solution, and impairing platelet aggregate formation under flow over collagen. In line with these findings, ex vivo collagen-induced platelet aggregation and aggregate formation on immobilized collagen under flow were also impaired in whole blood of donors that had consumed *Cannabis sativa*.

## 3. Procoagulatory Effects of Cannabinoids

While some reports in the literature have linked cannabinoid actions with hemorrhage, others illustrate an association between cannabinoid use and thromboembolic complications, suggesting procoagulatory effects of cannabinoids. In one case [58], the patient over a period of nine months had presented with recurrent thromboembolism manifested as acute bilateral renal infarcts, a left renal infarct, a pulmonary embolism (PE), and an ischemic stroke on four separate occasions following synthetic cannabinoid smoking. Her past medical history and family history were negative for potential risk factors of coagulopathy. Furthermore, her clinical investigation did not reveal any results that could explain her condition. She was started on anticoagulative therapy after her first thrombotic event and, despite being on a prophylactic daily aspirin (ASA), she developed repeated thromboembolism. The fact that each of these thromboembolic events was preceded by heavy smoking of cannabinoids is strongly indicative of a procoagulatory response triggered by these cannabinoids that possibly led to the activation of inflammatory pathways or coagulation pathways, albeit the exact mechanisms remain unknown.

Freeman et al. [59] reported two cases of young male and female siblings who presented independently with an acute ischemic stroke shortly after smoking synthetic cannabinoids. They have a history of smoking marijuana and tobacco, respectively; however, it has been found that they had smoked synthetic cannabinoids from the same batch prior to each of their presentations to the ER. A serum sample from the second patient was analyzed using liquid chromatography-tandem mass spectroscopy and it revealed the presence of JWH-018 but was negative for the other testable compounds (AM-2201, JWH-019, JWH-073, JWH-250). The patients had no significant medical history, and their family history was negative for blood clots, hypercoagulability, heart disease, stroke, or any risk factors of stroke. They had unremarkable laboratory investigation, but the presence of infarction with cerebral blood clots was confirmed by brain MRI and CT angiography for both patients. Two other cases of young females who presented with an ischemic stroke associated with synthetic cannabinoid use have also been reported [60]. While both patients developed an acute ischemic stroke soon after synthetic cannabis consumption, the patients have other risk factors for developing stroke including oral contraceptive use, a patent foramen ovale without venous thromboses, migraine with aura, smoking, and a family history of superficial thrombophlebitis. Whether their stroke was triggered by pre-existing risk factors or by their recent synthetic cannabinoid consumption remains to be determined.

Stupinski et al. [61] studied the impact of pre-injury chronic marijuana (THC) exposure on the development of thromboembolic complication (TEC) in adult trauma patients using a 2-year retrospective cohort analysis of the American College of Surgeons Trauma Quality and Improvement Program (ACS-TQIP) database. While trauma patients usually meet the criteria of Virchow’s triad (hypercoagulability, stasis, and endothelial injury) and, thus, are more susceptible to developing TEC, it has been found that trauma patients who chronically consumed the phytocannabinoids THC before their injury independently exhibited higher rates of venous TEC, namely, DVT (6.6% vs. 1.8%, *p* = 0.02) and PE (2.2% vs. 0.2%, *p* = 0.04) compared to their matching control groups with no prior chronic exposure to THC. Interestingly, pre-injury chronic exposure to THC had no significant effects on mortality rates (*p* = 0.28) nor on developing arterial TEC, namely, stroke (*p* = 0.24) and myocardial infarction (MI) (*p* = 0.35). Using a similar approach to study the impact of THC exposure on TEC in geriatric trauma patients (age > 65 years), researchers found that the rate of TEC was significantly higher in THC(+ve) geriatric trauma patients compared to THC(−ve) group (3.0% vs. 1.7%; *p* = 0.01). Furthermore, the THC(+ve) group had significantly higher rates of DVT (2.2% vs. 0.6%, *p* < 0.01) and PE (1.4% vs. 0.4%, *p* < 0.01) in comparison to control THC(−ve) patients [62].

Although the previous two studies have shown no association between cannabinoid use and developing MI in trauma patients, numerous other studies demonstrate a link between cannabinoid consumption and triggering myocardial ischemia or infarction. Mittleman et al. [63] conducted a case-crossover study with self-matched control to examine the association between frequency of MI onset and marijuana smoking within an hour before experiencing symptoms of acute MI. Results showed that, in the 60 min following marijuana smoking, the risk of MI onset is increased by 4.8 times over baseline (95% confidence interval, 2.4 to 9.5; *p* < 0.001), with a rapid decline in the risk thereafter. Consistent with these results, several cases reported an incidence of myocardial ischemia or infarction that is temporally associated with cannabinoid consumption [64,65,66,67]. The potential mechanisms underlying the transient elevated risk of MI post cannabinoid smoking are not fully understood; however, cannabinoid smoking is associated with rapid absorption of cannabinoids through the lung circulation, and the concentration of these compounds can reach peak levels in the systemic circulation within 30 min after smoking. In addition, previous studies indicate that cannabinoids (marijuana or synthetic ligands) can influence cardiovascular autonomic nervous system activity via CB1 receptor activation, resulting in a wide range of acute hemodynamic effects that largely depend on the cannabinoid dose, route of administration, duration of the use, and individual sensitivity. In this context, hypotension, increased heart rate by 20–100%, and decreased myocardial contractility have been documented [68,69,70]. These effects may increase the myocardium workload, thereby increasing the cardiac oxygen demand with unmatched oxygen supply. Moreover, cannabinoid smoking increases the production of carboxyhemoglobin [71], which, along with the compromised oxygen supply, can further impair the myocardial oxygen balance. Generation of reactive oxygen species (ROS) and subsequent endothelial dysfunction have also been reported with CB1 receptor activation [70]. Altogether, these acute effects may associate with transient ischemic/reperfusion changes with increased oxidative stress at the cardiovascular tissue levels that may, ultimately, lead to the activation of coagulative pathways causing MI in susceptible individuals.

In vitro studies conducted to examine the effects of endogenous cannabinoids on platelet function may give insights into possible mechanisms underlying the cannabinoid-induced procoagulatory responses seen with the previously reported thromboembolism cases associated with cannabinoid use, although, as indicated above, other mechanisms might be implicated. A study examined the effects of the main endocannabinoids AEA, 2-AG, and virodhamide on platelet aggregation in human blood and platelet-rich plasma (PRP) samples using multiple electrode aggregometry showed that both 2-AG and virodhamide stimulated platelet aggregation in blood, and induced shape change and adenosine triphosphate (ATP) release that was followed by platelet aggregation in PRP, whereas AEA (600 µM) was inactive. In addition, the stimulatory effects of 2-AG and virodhamide on platelet aggregation in blood and PRP were dose-dependent. The synthetic cannabinoids ACEA, a CB1 agonist, and JHW015, a CB2 agonist, showed neither a stimulatory nor inhibitory effect on platelet aggregation. The platelet aggregation induced by 2-AG and virodhamide was inhibited by ASA, a COX-1 inhibitor, daltroban, a specific TXA_2_-receptor antagonist, and JZL184, a MAGL inhibitor, suggesting that this aggregation resulted from the degradation of these endocannabinoids into free AA and its metabolite TXA_2_, rather than direct CB1/CB2 activation [72]. Baldassarri et al. [73] have also found that the 2-AG-induced platelet activation in gel-filtered platelets is independent of CB1/CB2 stimulation, as they could not detect the presence of CB1 and CB2 mRNA nor their proteins on the platelet surface. It has been demonstrated that the platelet activation was triggered by a robust release of TXA_2_ from these platelets leading to cytoplasmic Ca^2+^ release, granule secretion, and platelet aggregation. The 2-AG-induced platelet aggregation, however, was not inhibited by MAGL inhibitors, which prevent the degradation of 2-AG into AA and TXA_2._

On the other hand, a study on washed human platelets demonstrated a dose-dependent platelet activation by AEA (250–1300 µM) resembling the effects induced by AA. However, unlike the AA-induced platelet activation, the activation triggered by AEA was not inhibited by ASA. Furthermore, PMSF, an inhibitor of FAAH that degrades AEA into AA, did not affect platelet activation induced by AEA, suggesting that this activation is independent of the AA pathway with no evidence to support a CB1/CB2 activation-dependent mechanism [74]. Another in vitro study, however, was able to show the existence of both CB1 and CB2 cannabinoid receptor expression on human platelet surface using Western blot. It also investigated the effects of THC (final concentrations 10^−7^ to 10^−5^ M) on platelet activation. Whole blood flow cytometric analyses revealed that THC increased the expression of activated platelet surface markers: fibrinogen receptor (glycoprotein IIb-IIIa) and P selectin in a dose-dependent manner [75]. Together, the results of this study may suggest a procoagulatory effect of cannabinoids that is dependent on cannabinoid receptor activation. A more recent study has also confirmed the presence of CB1 and, to a lesser extent, CB2 receptor proteins intracellularly in human platelets using Western blot, ELISA, and confocal fluorescence microscopy [76]; however, further in vivo studies are needed to confirm the potential involvement of CB1/CB2 stimulation in platelet activation and blood coagulation.

## 4. Discussion

As illustrated in the previous two sections, members of all classes of cannabinoids (synthetic, plant-derived, and endogenous), despite being vastly different in their structure, receptor affinity, potency, and metabolism can have pro- or anti-coagulatory (Table 1). The reported variations in the coagulatory effects of a particular cannabinoid often depends on the experimental conditions under which the cannabinoid was studied. In the case of synthetic cannabinoids, contamination with other substances might largely be responsible for the observed anti-/pro-coagulatory effects. Nonetheless, plant-derived cannabinoids have shown interactions with warfarin, a well-known vitamin K antagonist that is commonly prescribed as an anticoagulant for many clinical indications including stroke prevention and DVT treatment and other cardiovascular conditions. Studies have indicated that both phytocannabinoids, Δ^9^-THC and CBD, are potential inhibitors for the enzymatic activity of the cytochrome P450 enzyme CYP2C9 [77,78,79], which is the primary metabolic site where the S-enantiomer of warfarin, the part of the warfarin medication that exhibits the most potent anticoagulative effect, undergoes significant oxidative metabolism in the liver [79]. Therefore, the anticoagulative effect of warfarin could potentially be potentiated using these cannabinoids. With the recent increase in the interest in exploring the clinical potential of cannabinoids and the medicinal use of marijuana, clinicians should be aware of such drug–drug interactions that affect blood coagulation like the one reported with an FDA-approved CBD-based drug for treating intractable epilepsy, (Epidiolex, Greenwich Biosciences) [50]. In addition to the indirect anticoagulatory effects of phytocannabinoids, some of them indeed have been shown to exhibit anticoagulatory effects in pre-clinical studies by inhibiting thrombin activity [12,52] or platelet aggregation [53,57]. It seems, however, that consuming phytocannabinoids (or sometimes synthetic ligands) by smoking is likely to be associated with procoagulatory effects [58,59,63,66,67]; whether smoking itself is responsible for inducing the procoagulatory effects of these cannabinoids remains to be elucidated. Reported coagulatory studies of endogenous cannabinoids have been mainly done in vitro and, except for one study that demonstrated an anticoagulatory effect of AEA by inhibiting platelet aggregation [57], most of these studies support a procoagulatory effect through stimulating platelet activation and aggregation, although the exact mechanisms by which this effect occurs are still controversial [72,73,74].

The presented studies in this review do show the potential of different cannabinoids in modulating blood coagulation. Both anticoagulatory and procoagulatory effects could be beneficial in clinical use, as coagulopathies can manifest as abnormal clot formation or hemorrhage. However, the heterogenicity of the studies makes it difficult to conclude a statement on the coagulatory effects of cannabinoids. We are still far away from translating these preliminary findings into clinical practice. Careful designing of further experiments both in vitro and in vivo is needed to understand the conditions under which pro- or anti-coagulatory effects of these cannabinoids may predominate, as well as the underlying mechanisms of each of these effects. Further research is also needed to overcome the undesired psychoactive effects which might be associated with cannabis-based drugs. 

## 5. Conclusions

In summary, while the literature displays controversial findings on the coagulatory effects of cannabinoids, there is strong evidence that supports the presence of crosstalk between the ECS and the coagulation system. Multiple mechanisms might be implicated in the coagulatory effects seen with different cannabinoids and these variations in the effects may also be influenced by other factors including the dose, route of administration, duration of the use, and individual sensitivity, as well as the health/disease state of the subject, as the synthesis and expression of CB1/CB2 receptors is regulated on demand. Both CB1 and CB2 receptors have been identified on human platelet, suggesting potential roles for cannabinoids in modulating platelet function and blood coagulation. Further studies are needed to understand the cannabinoid effects on blood coagulation and the association between the ECS and the coagulation system, which would help in developing new therapeutic strategies for coagulopathies.

## Figures and Tables

**Table 1:** Overview of the reported coagulatory effects of various classes of cannabinoids with potential underlying mechanisms.

Cannabinoid	Anticoagulatory Effects with Potential Underlying Mechanisms	Reference
**Synthetic**		
Synthetic Cannabinoids	Indirect anticoagulatory effect → contamination of cannabinoid with brodifacoum (Vitamin K antagonist).	[44,45,46,47,48]
**Plant-derived**		
THC, CBD, cannabis	Drug–drug interactions → cannabinoids may potentiate the anticoagulative effect of warfarin in patients taking warfarin therapy through cytochrome P450 interaction.	[49,50,51]
Cannabis extract	Prolongation of thrombin-induced clotting time in diabetic Wistar rats.	[12]
Cannabis extract, THC, CBN	Inhibition of thrombin-induced clotting formation in vivo and in vitro.	[52]
Olivetol, CBG, CBN, CBD, THC	Inhibition of human and rabbit platelet aggregation.	[53]
Cannabis smoking	Diffuse alveolar hemorrhage and hemoptysis (unknown mechanisms)	[54,55,56]
*Cannabis sativa*	Impairment of collagen-induced platelet aggregation and aggregate formation on immobilized collagen under flow ex vivo.	[57]
**Endogenous**		
AEA	Inhibition of platelet aggregation and aggregate formation under flow over collagen in vitro through inhibiting P selectin expression and limiting glycoprotein IIb/IIIa activation.	[57]
**Cannabinoid**	**Procoagulatory Effects with Potential Underlying Mechanisms**	**Reference**
**Synthetic**		
Synthetic cannabinoid smoking	Repeated thromboembolic events with possible activation of inflammatory or coagulative pathways.	[58]
Synthetic cannabinoid smoking (JWH-018)	Acute ischemic stroke in patients with no prior risk factors for stroke (unknown mechanisms).	[59]
Synthetic cannabinoid use	Acute ischemic stroke with other prior risk factors for developing stroke.	[60]
**Plant-derived**		
Chronic THC exposure	Increase in the risk of developing venous thromboembolic complications in adult and geriatric trauma patients (unknown mechanisms).	[61,62]
Acute marijuana smoking	Increase in the risk of M.I by 5 times over baseline during the 60 min following acute marijuana smoking. Potential mechanisms: Activation of CB1 receptors with increased oxidative stress at the cardiovascular tissue level that may ultimately lead to the activation of coagulative pathways.	[63,68,69,70,71]
THC	Stimulation of platelet activation via CB1/CB2-dependent mechanism through increasing fibrinogen receptor (glycoprotein IIb-IIIa) and P selectin expression.	[75]
**Endogenous**		
2-AG, Virodhamide	Stimulation of platelet aggregation in human blood and PRP samples through degradation of these endocannabinoids into AA metabolite (TXA_2_).	[72]
2-AG	Stimulation of platelet activation accompanied by a robust TXA_2_ release from these platelets leading to cytoplasmic Ca^2^+ release, granule secretion, and platelet aggregation.	[73]
AEA	Stimulation of platelet activation through a mechanism independent of the AA pathway.	[74]
